# Wnt3a signal pathways activate MyoD expression by targeting *cis*-elements inside and outside its distal enhancer

**DOI:** 10.1042/BSR20140177

**Published:** 2015-03-18

**Authors:** Yu Chih Pan, Xiao Wen Wang, Han Feng Teng, Yi Ju Wu, Hsuan Chia Chang, Shen Liang Chen

**Affiliations:** *Department of Life Sciences, National Central University, 300 Jhongda Road, Jhongli 32001, Taiwan

**Keywords:** β-catenin, muscle, MyoD, myogenesis, Pax7, Wnt3a, BAC, bacterial artificial chromosome, CMB, confluent myoblast, DE, distal enhancer, DMEM, Dulbecco's modified Eagle's medium, dpc, day(s) postcoitum, DRR, distal regulatory region, Fgf, fibroblast growth factor, Gapdh, glyceraldehyde-3-phosphate dehydrogenase, GSK3β, glycogen synthase kinase 3β, HEK, human embryonic kidney, HRP, horseradish peroxidase, JNK, c-Jun N-terminal kinase, LEF, lymphoid enhancer factor, MAPK, mitogen-activated protein kinase, MRF, myogenic regulatory factor, MT, myotube, NFAT, nuclear factor of activated T-cells, PBST, PBS containing 0.5% Tween 20, P6P, proximal 6 kb promoter, PRR, proximal regulatory region, qRT-PCR, quantitative reverse transcription–PCR, Shh, Sonic Hedgehog, SKM, skeletal muscle, T_3_, 3,3′,5-tri-iodothyronine, TKE, thymidine kinase promoter and enhancer, TRE, T_3_-response element, WRE, Wnt-response element

## Abstract

Wnt proteins are secreted cytokines and several Wnts are expressed in the developing somites and surrounding tissues. Without proper Wnt stimulation, the organization of the dermomyotome and myotome can become defective. These Wnt signals received by somitic cells can lead to activation of Pax3/Pax7 and myogenic regulatory factors (MRFs), especially Myf5 and MyoD. However, it is currently unknown whether Wnts activate Myf5 and MyoD through direct targeting of their *cis*-regulatory elements or via indirect pathways. To clarify this issue, in the present study, we tested the regulation of MyoD *cis*-regulatory elements by Wnt3a secreted from human embryonic kidney (HEK)-293T cells. We found that Wnt3a activated the *MyoD* proximal 6.0k promoter (P6P) only marginally, but highly enhanced the activity of the composite P6P plus distal enhancer (DE) reporter through canonical and non-canonical pathways. Further screening of the intervening fragments between the DE and the P6P identified a strong Wnt-response element (WRE) in the upstream −8 to −9k region (L fragment) that acted independently of the DE, but was dependent on the P6P. Deletion of a Pax3/Pax7-targeted site in the L fragment significantly reduced its response to Wnt3a, implying that Wnt3a activates the L fragment partially through Pax3/Pax7 action. Binding of β-catenin and Pax7 to their target sites in the DE and the L fragment respectively was also demonstrated by ChIP. These observations demonstrated the first time that Wnt3a can directly activate MyoD expression through targeting *cis*-elements in the DE and the L fragment.

## Abstract 2

We found that Wnt3a can directly activate MyoD expression through targeting *cis*-elements in the distal enhancer and in the upstream −8 to −9k region. A novel Pax3/Pax7-involved pathway and both canonical and non-canonical Wnt pathways are involved in this activation.

## INTRODUCTION

Trunk skeletal muscle (SKM) cells in vertebrates are derived from precursors in the embryonic somites, and a subset of somitic cells become myogenic stem cells after being specified by local signals to express Pax3 and Pax7 [1]. They are further confined within the myogenic lineage by the expression of either MyoD or Myf5 that drives them to become myoblasts. Upon the stimulation of differentiation signals, myoblasts start expressing myogenin and Mef2c that co-operatively drive the formation of multinucleated myotubes, the expression of contractile proteins and cell cycle exit. Myogenic stem cells are gradually confined to the periphery of mature myotubes during late embryogenesis to become satellite cells that are responsible for postnatal muscle growth and repair [2].

In mice, the expression of Myf5 and MyoD initiates at 8.5 and 10.5 days postcoitum (dpc) respectively, whereas the order of their expression is reversed in chicken [3–5]. Mice carrying a *Myf5* homozygous mutation die at birth due to the absence of the distal parts of the ribs, which results in the inability to breathe [6]; however, the expression levels of other myogenic regulatory factors (MRFs) in *Myf5*-null mice appear to be normal. Surprisingly, *MyoD*-null mice are fully viable and show no obvious muscle abnormalities [7]. Since mice carrying null mutations in either the *Myf5* or the *MyoD* gene have apparently normal SKM, it raises the possibility that these two myogenic factors are functionally redundant in myogenesis. This speculation was confirmed when mice carrying null mutations in both *Myf5* and *MyoD* loci were found to have a complete absence of SKM and desmin-expressing myoblast-like cells [8]. These observations suggest that either Myf5 or MyoD is required for the determination of skeletal myoblasts or their propagation, or both, during embryonic myogenesis.

It was surprising to find that *MyoD*-null mice shown severe deficiency in regenerative capacity after injury, suggesting that MyoD plays critical roles in regulating the postnatal myogenic program of satellite cells [9]. Quiescent satellite cells express low or undetectable levels of MyoD, but increase their MyoD expression upon receiving activation signals for proliferation. High MyoD levels repress satellite cell self-renewal and drive their myogenic differentiation or apoptosis [10]; therefore the level of MyoD plays critical roles in the regulation of satellite pool replenishment and the ability to regenerate damaged tissue.

The expression pattern of human MyoD during embryogenesis can be recapitulated by the upstream 24 kb flanking sequence, and several major *cis*-elements within this region regulating MyoD expression have been identified [11]. A distal enhancer (DE) centred at −20 kb, when combined with a −2.5k proximal promoter, can also recapitulate human MyoD expression *in vivo*, demonstrating its critical role in determining the spatiotemporal expression of human MyoD [12]. A proximal 6 kb promoter (P6P) sequence of the mouse *MyoD* gene containing a proximal regulatory region (PRR) and a distal regulatory region (DRR) are sufficient to activate muscle-specific expression of MyoD *in vitro* and *in vivo*, but leaky expression in the central nervous system and delayed expression in limb buds and bronchial arches were observed [13,14]. Currently, it is unknown whether the combination of mouse DE and P6P sequences can faithfully drive MyoD expression *in vivo*.

The Wnt protein family consists of 19 secreted paracrine glycoproteins that act by binding to Frizzled receptors on target cell plasma membrane [15]. Multiple intracellular signalling pathways can be activated by Wnt–receptor interaction, of which inactivation of glycogen synthase kinase 3β (GSK3β) leading to the cytoplasmic accumulation and nuclear import of β-catenin is the best characterized and is known as the canonical Wnt pathway. Nuclear β-catenin dimerizes with T-cell factor (TCF)/lymphoid enhancer factor (LEF) on target gene promoters to drive their transcription to mediate cellular responses to Wnt signals. In addition, Wnt–receptor-activated pathways can also activate cytoplasmic c-Jun N-terminal kinase (JNK) and nuclear factor of activated T-cells (NFAT) to allow them to translocate into the nucleus to activate target genes [16].

Several Wnts are expressed in somites and surrounding tissues and are implicated in myogenesis. Wnt1, Wnt3a and Wnt4 are expressed in the dorsal neural tube; Wnt4, Wnt6 and Wnt7a are expressed in the dorsal ectoderm; Wnt11 is expressed in the epaxial dermomyotome [15,17]. These Wnt signals received by somitic cells can lead to activation of Pax3/Pax7 and/or MRFs. Without proper Wnt stimulation, the organization of the dermomyotome and myotome can become defective [18]. A combination of Wnts in the dorsal neural tube co-operates with a low level of Sonic Hedgehog (Shh) to induce the expression of Myf5 in epaxial myotome. At the same time, MyoD is induced in the hypaxial myotome by the ectodermally derived Wnts and the lateral plate mesoderm-released bone morphogenetic protein 4 (BMP4) and fibroblast growth factor 5 (Fgf5). Neural tubes of later stages (>10.5 dpc) can also induce MyoD expression in epaxial myotomes, indicating the transition in either the competence of the myotomic cells to Wnt induction or in the composition of neural-tube-derived Wnts [19].

In resting adult muscle, Wnt5a, Wnt5b, Wnt7a and Wnt4 are expressed, and the expression of the first three Wnts increases in the early phase of muscle injury. In the later stages, Wnt7b and Wnt3a become detectable [20]. Application of Wnt3a in the early phase of muscle regeneration induced premature differentiation of progenitor cells, thereby leading to depletion of the satellite cell pool. In aged mice, systemic Wnt signals induce myogenic stem cells into fibrogenic cells and thus reduce the regenerative capability of SKM in aged subjects [21]. Therefore the subtle regulation of satellite cells by Wnt in adult SKM plays critical roles in SKM regeneration and aging.

The expression of Myf5 and MyoD has been shown to be activated by Wnts differentially: Wnt1 and Wnt7a preferentially activate Myf5 and MyoD respectively, whereas Wnt4, Wnt5 and Wnt6 activate both MRFs at an intermediate level; the expression of MyoD was also found to be enhanced by Wnt3a during directed myogenic differentiation of P19 cells [22–24]. However, it is currently unknown whether Wnts activate MyoD through direct targeting of their *cis*-regulatory elements or via indirect pathways. To clarify this issue, detailed study on the *cis*-elements of *MyoD* gene regulatory regions is required.

## MATERIALS AND METHODS

### Plasmids

The *MyoD* promoter region −5870 to +95 was PCR-amplified using primers (NCUTC021003/NCUTC021004) from MD6.8-lacZ (a gift from Dr Atsushi Asakura) and inserted into the KpnI/NheI sites of pStable-luc vector [25] to generate pStable-MyoD 6.0-luc reporter. A linker sequence (5′-GTACGAATTCACGCGTGTAC-3′) containing the EcoRI/MluI sites was inserted into the KpnI site of the above plasmid to generate pStable-MyoD 6.0-adaptor-luc reporter, which was further modified to become pStable-MyoD 6.0-enhance-luc (PE) by inserting the distal enhancer (−25277 to −20781) amplified from mouse genomic bacterial artificial chromosome (BAC) clone (RP23-284P22) into the EcoRI site. Genomic fragments between the promoter and the core enhancer were PCR-amplified from the above BAC clone using the primers listed in Supplementary Table S1 and inserted into the PE reporter for screening their involvement in Wnt3a response. *Wnt3a* coding sequence was released from PGK-puro-*Wnt3a* (a gift from Dr Ilona Skerjanc) by BamHI/XhoI and inserted into XhoI (blunted) site of the pPyCAG-IP vector for creating pPyCAG-IP-Wnt3a expression vector. The coding sequences of β-Catenin Δ90 and Δ151 were PCR-amplified and inserted into pCMV-Flag vector to create C-terminally FLAG-tagged proteins. Then, both coding sequences were released and inserted into the EcoRI site of pCDNA3.1 and the XhoI site of pPyCAG-IP vectors to create mammalian expression vectors that can be stably integrated into chromosomes. The expression vectors of both dominant-negative NFAT and JNK1 were gifts from Dr Roger Davis [26].

### Stable cloning of reporters and promoter assay

Proliferating C2C12 cells were kept at low confluence in Dulbecco's modified Eagle's medium (DMEM) supplemented with 20% (v/v) FBS. For inducing myotube formation, confluent myoblasts were kept in differentiation medium (DMEM supplemented with 25 nM insulin and 5 mM LiCl) for 4–6 days, before being harvested for staining and photographing. The stable cloning of pStable-luc based reporter into C2C12 was as described previously [27]. Briefly, aliquots (approximately 5 μg) of pStable-MyoD 6.0-luc (or other derived reporters) DNA were mixed 1:5 with Lipofectamine™ (Invitrogen) in Hepes buffer (20 mM Hepes, pH 7.0, 187 mM NaCl, 5 mM KCl, 0.7 mM Na_2_HPO_4_ and 5.5 mM dextrose) in 1.5 ml tubes and incubated at room temperature for 10–15 min to allow DNA and liposome complexes to form. Then, the mixture was transferred to cells grown in 6-mm-diameter Petri dishes and the transfection was allowed to proceed overnight before the medium was replaced by fresh medium. G418 (800 μg/ml) was added to the medium 48 h after transfection and the selection was allowed to proceed for 2–3 weeks until monoclonal colonies appeared. Colonies were pooled together to form polyclonal population and used for promoter assay. The stable clones of cells carrying other *MyoD cis-*element-driven reporters were similarly generated. Human embryonic kidney (HEK)-293T cells were kept in DMEM supplemented with 10% (v/v) FBS. For establishing stable clones of HEK-293T-Wnt3a cells, pPyCAG-IP-Wnt3a was transfected into HEK-293 cells and selected with puromycin (3 μg/ml) as above.

For transient transfection assay, C2C12 cells were split into 12-well plates and allowed to grow to 70–80% confluence. Then, cells were co-transfected with promoter-driven reporters (0.67 μg/well) and expression vectors (0.17 μg/well) for 16 h as described above for stable cloning of reporters, and then treated with pathway inhibitors or other compounds for 24 h before being harvested for determining their luciferase activity using the Bio-Tek Clarity 2 luminometer. All reactions were carried out in triplicate and repeated at least three times.

### Somite explant culture

Embryos of 10.5 dpc were dissected out from the uterus and washed extensively with PBS. Then, visceral mass, head, tail and limbs were carefully removed under a dissection microscope. Somites were cultured in control (pPyCAG-IP) or Wnt3a-containing medium for 48 h before being harvested for isolating total RNA. The animal experiments had been approved by the Institutional Animal Care and Use Committee (IACUC) of National Central University.

### Western blot and quantitative reverse transcription–PCR (qRT-PCR)

The RNA extraction and qRT-PCR procedures have been described in our previous papers [25,27]. Briefly, total RNA was extracted from the C2C12 cells using TRIzol® (Life Technologies) according to the supplier's instructions. Then, the first strand of cDNA was synthesized using the Superscript III kit (Life Technologies). qRT-PCR was performed in 25 μl reaction volumes containing 5 μM forward/reverse primers, SYBR Green reaction mixture (Applied Biosystems) in an ABI 7300 sequence detection system. Glyceraldehyde-3-phosphate dehydrogenase (Gapdh) was used as an internal control and amplified in the same PCR assay. All PCR amplicons cover at least one intron to avoid amplification of genomic DNA in the PCR and primer sets used for each gene are listed in Supplementary Table S2.

The protocol for Western blotting has been described previously [25]. Briefly, aliquots of total lysate (50 μg) in RIPA buffer supplemented with protease inhibitors and phosphatase inhibitors were separated by SDS/PAGE (10% gels) before being blotted on to a PVDF membrane (Pall FluoroTrans W membrane). The PVDF membranes were washed extensively with PBS containing 0.5% Tween 20 (PBST) and blocked with 5% (w/v) dried skimmed milk powder in PBST. Primary antibody was diluted 1:1000 in blocking solution and incubated with the blot at 4°C for overnight. After several washes with PBST, horseradish peroxidase (HRP)-conjugated secondary antibody (1:10000 dilution) was added and incubated at room temperature for 1 h. After extensive washing, the HRP signal was detected by a chemiluminescence kit (GE Healthcare). The antibodies against Wnt3a and Pax7 were purchased from Cell Signaling Technology (#C64F2) and Neuromics (#MO15020) respectively.

### ChIP assay

The detailed procedure of the ChIP assay has been described in our previous papers [25,28,29]. Briefly, C2C12 cells were washed, fixed in formaldehyde (1%, w/v), and sonicated to shear chromatin. Specific antibody was added to the cleared lysate and the binding was allowed to proceed at 4°C overnight before *Staphylococcus aureus* Protein A (Sigma, #P7155) was added to capture the immune complex and then washed extensively. Then, the immune complex was eluted and the released DNA was extracted with phenol/chloroform twice and further purified using a PCR purification Kit (Geneaid). The primer sets used in the ChIP assay are listed in Supplementary Table S3.

## RESULTS

### Wnt3a activate MyoD expression in myoblasts and somite explants

**Figure 1:**
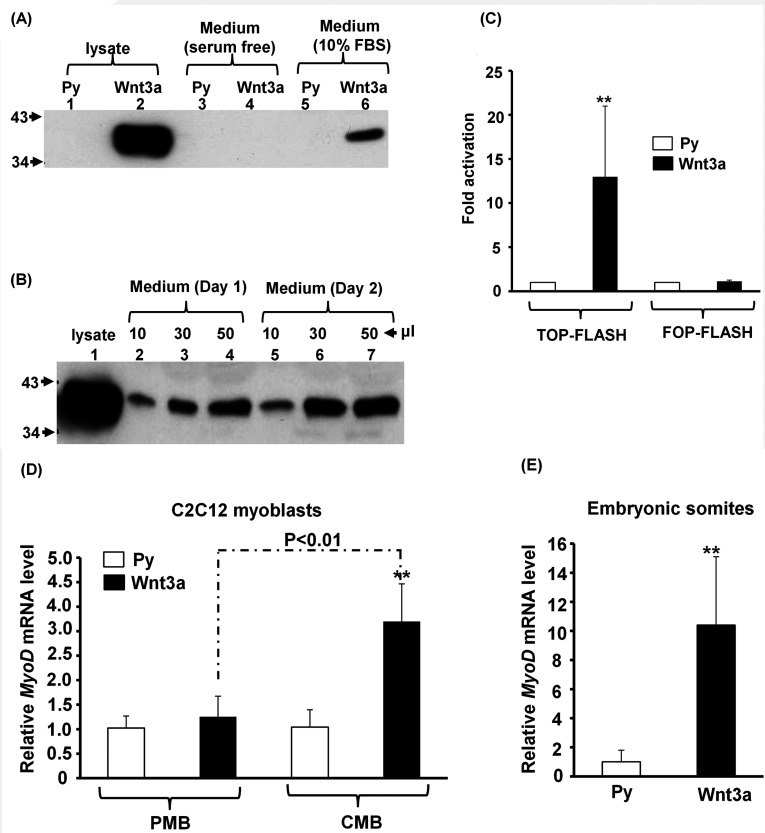
Establishment of Wnt3a-secreting HEK-293T stable clones (**A**) The Wnt3a levels (in 20 μl of medium) accumulated in the last 24 h of HEK-293T-Py and -Wnt3a stable clones cultured in serum-free medium or regular medium (with 10% FBS) was determined by Western blotting. (**B**) Growth medium (containing 10% FBS) from HEK-293T-Wnt3a cells was collected on day 1 and day 2 after cells became confluent. Different volumes of this medium were then subjected to Western blotting using anti-Wnt3a antibody. Total lysate (50 μg) from cells kept in medium containing 10% FBS was used as positive control. (**C**) Either TOPflash or FOPflash was transiently transfected into C2C12 myoblasts and then treated with medium from HEK-293T-Py or -Wnt3a cells for 24 h before being harvested for determination of luciferase activity. The activity in cells treated with Py medium was set as 1-fold activation. **P<0.01 compared with Py medium. (**D** and **E**) Wnt3a activates MyoD expression in C2C12 cells (**D**) and in somite explants (**E**). The expression level of MyoD in proliferating (PMB) and confluent (CMB) C2C12 cells and in cultured somite explants treated with or without Wnt3a medium was determined by qRT-PCR. The MyoD level in cells or somites treated with Py medium was set as 1-fold. **P<0.01 compared with Py medium.

Wnt3a is post-translationally modified and these modifications are critical to its function; therefore recombinant proteins expressed in bacteria might not be an effective source for treating mammalian cells. To ensure proper post-translational modifications, the coding sequence of Wnt3a was overexpressed in HEK-293T cells by super-transfection, in which the vector pPyCAG-IP (Py hereinafter) carrying the Wnt3a coding sequence could replicate extensively in HEK-293T cells to express high levels of Wnt3a [30]. The expression of Wnt3a in HEK-293-Wnt3a cell lysates and various culture media was determined by Western blotting, and we found that serum was critical for its accumulation in the medium (Figure 1A). Wnt3a protein in the medium reached its highest level 2 days after cells became confluent (Figure 1B). Medium harvested at this time point was routinely collected as the source of Wnt3a (denoted as Wnt3a medium) and it could strongly activate the Wnt signalling pathway reporter TOPflash (Figure 1C), indicating that Wnt3a secreted from HEK-293T-Wnt3a cells is functional.

To examine the effect of Wnt3a on MyoD expression, C2C12 myoblasts were kept in control (Py) or Wnt3a-containing medium and their *MyoD* mRNA level was determined by qRT-PCR. We found that Wnt3a had no effect on the *MyoD* mRNA level in proliferating C2C12 myoblasts, but enhanced its expression significantly in confluent myoblasts (Figure 1D), suggesting that the Wnt3a signalling pathway promotes transcription of the *MyoD* gene in a differentiation stage-dependent manner. The same activation effect on the *MyoD* mRNA levels of cultured somite explants from mouse embryos of 10.5 dpc was also observed (Figure 1E), demonstrating that the Wnt3a prepared in the present study can also activate somitic cells to express MyoD.

### *Cis*-elements in distal enhancer mediate Wnt3a response

**Figure 2:**
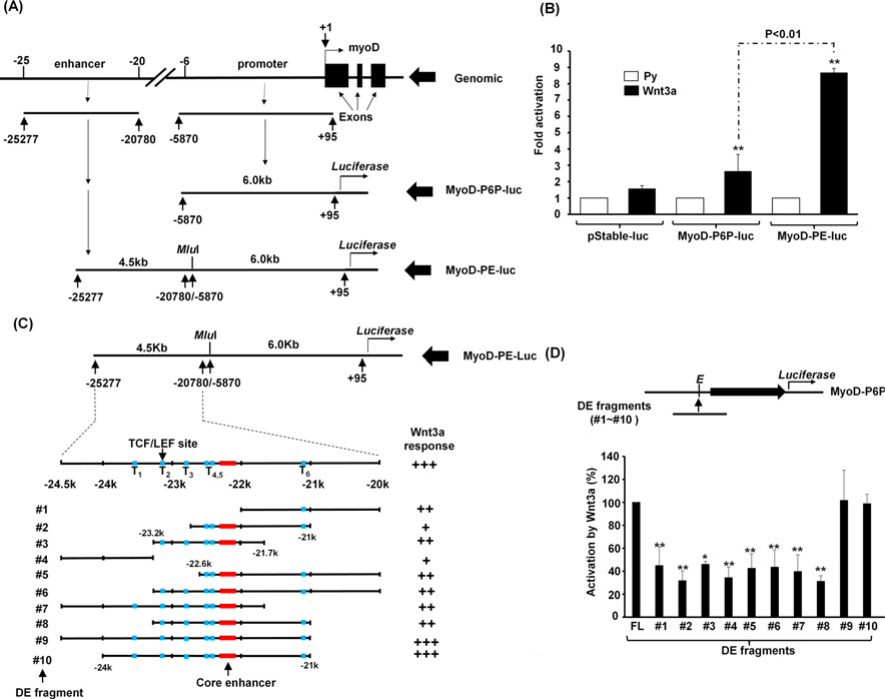
Wnt3a activates MyoD expression via distal enhancer (**A**) Schematic diagrams of the construction of *MyoD* promoter-driven reporters. The genomic organization of the *MyoD* gene is shown on the top. Reporters driven by the proximal 6.0k promoter (P6P) or P6P plus the distal enhancer were named MyoD-P6P-luc and MyoD-PE-luc respectively. An MluI site between the promoter and enhancer allows insertion of other genomic fragments. The activation of both reporters by Wnt3a is shown in (**B**). (**C**) Diagrams showing various MyoD-PE-luc-derived reporters in which the DE was replaced by various individual DE fragments. Multiple putative TCF/LEF target sites (T1–T6, blue boxes) in the DE and the conserved core enhancer (258 bp, orange box) are indicated. The responses of these reporters to Wnt3a are shown in (**D**). *P<0.05 and **P<0.01 compared with full-length (FL) DE. All reporters used were stably carried by C2C12 cells.

It was of interest to decipher how Wnt3a activates *MyoD* transcription and to clarify whether it targeted the *MyoD* gene directly or through activation of other upstream regulators. To this end, C2C12 myoblasts stably carrying the P6P (−5870 to +95) -driven reporter (MyoD-P6P-luc) or another composite reporter, MyoD-PE-luc, containing P6P plus the DE (−25277 to −20780) (Figure 2A) were treated with Wnt3a medium. We found that Wnt3a marginally, but significantly, activated MyoD-P6P-luc activity (Figure 2B); however, Wnt3a strongly activated MyoD-PE-luc, suggesting that one or multiple Wnt-response elements (WREs) are located within the DE.

Using a bioinformatics tool (http://www.genomatix.de/matinspector), we found multiple putative TCF/LEF-binding sites (T1–T6, matrix similarity >0.9) in the DE. To identify and confirm that these putative WREs in the DE were responsible for the Wnt3a response, the DE was first separated into four fragments of approximately 1.5 kb (Figure 2C, fragments 1–4). Unfortunately, none of these fragments fully retained Wnt3a-induced activation (Figure 2D), suggesting that multiple weak WREs scattered within the DE region are responsible for the strong Wnt3a activation. Serial deletion mutants of DE region were generated later to identify the minimal WRE (Figure 2C), and we found that a region of 3 kb (fragment 10, −24 to −21 kb) in DE was required to retain the full Wnt3a response (Figure 2D). Deletion of sequence at either the 5′ end (fragment 8) or the 3′ end (fragment 7) significantly reduced its Wnt3a response, compared with fragment 10. These results suggest that the outer two flanking TCF/LEF-binding sites (T1 and T6) in DE play an essential role in its response to Wnt3a.

### Multiple pathways are employed to activate distal enhancer

**Figure 3:**
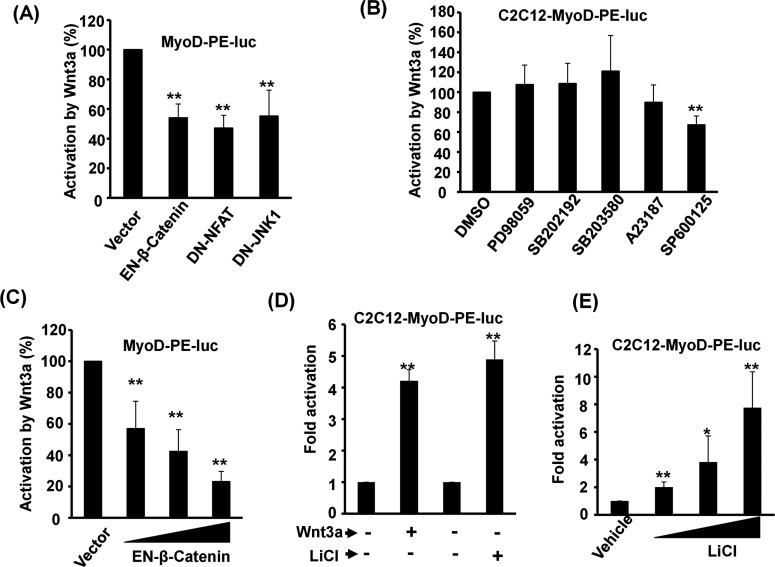
Wnt3a activates MyoD expression through multiple pathways (**A**) MyoD-PE-Luc was transiently co-transfected with vectors expressing dominant-negative (DN) mutants of Wnt pathway downstream effectors into C2C12 cells treated with or without Wnt3a. Luciferase activity was determined 24 h after transfection and their relative activation by Wnt3a is shown. (**B**) C2C12-MyoD-PE-luc stable clones were treated with Wnt3a in the presence or absence of MAPK inhibitors or A23187 for 48 h, and their relative activation by Wnt3a is shown. (**C**) Increasing amount of engrail–β-catenin (EN-β-catenin) was co-transfected with MyoD-PE-Luc into C2C12 cells as described in (**A**). (**D** and **E**) C2C12-MyoD-PE-luc stable clones were treated with Wnt3a or LiCl or neither (**D**) or increasing amounts of LiCl alone (**E**) for 48 h, and their activity relative to Py or vehicle control is shown. *P<0.05 and **P<0.01 compared with vector or vehicle control.

As the Wnt signal could be transduced by three independent pathways, it was of interest to know whether Wnt3a was preferentially transduced by any one of them. By co-expressing dominant-negative end effectors of these pathways, namely β-catenin, NFAT and JNK, we found that all dominant-negative effectors partially repressed the Wnt3a response (Figure 3A), suggesting that all three pathways were employed by Wnt3a to activate MyoD-PE-luc. The involvement of these pathways was further tested by applying inhibitors of mitogen-activated protein kinase (MAPK) pathways and Ca^2+^ ionophore A23187 to this assay system. The specific inhibitor of the JNK pathway, SP600125, was the only inhibitor found to partially block Wnt3a-activated MyoD-PE-luc (Figure 3B), confirming the involvement of the JNK pathway. In contrast, the activation of the Ca^2+^ signalling pathway by Ca^2+^ ionophore A23187 failed to alter either the basal activity or the Wnt3a response of MyoD-PE-luc (Figure 3B), suggesting that NFAT, as reported previously [31], might be indirectly activated by GSK3β instead of the Ca^2+^ signalling pathway. The involvement of the canonical pathway was analysed further and we found that the engrail–β-catenin fusion protein that functions as a dominant-negative homologue of β-catenin [24], repressed MyoD-PE-luc dose-dependently (Figure 3C). LiCl, the inhibitor of GSK3 enzymes, also activated MyoD-PE-luc to a level similar to that triggered by Wnt3a and in a dose-dependent manner (Figures 3D and 3E), demonstrating further the involvement of the canonical Wnt pathway in the activation of *MyoD* gene by targeting its DE.

### Overexpression of constitutively active β-catenin enhances MyoD expression

**Figure 4:**
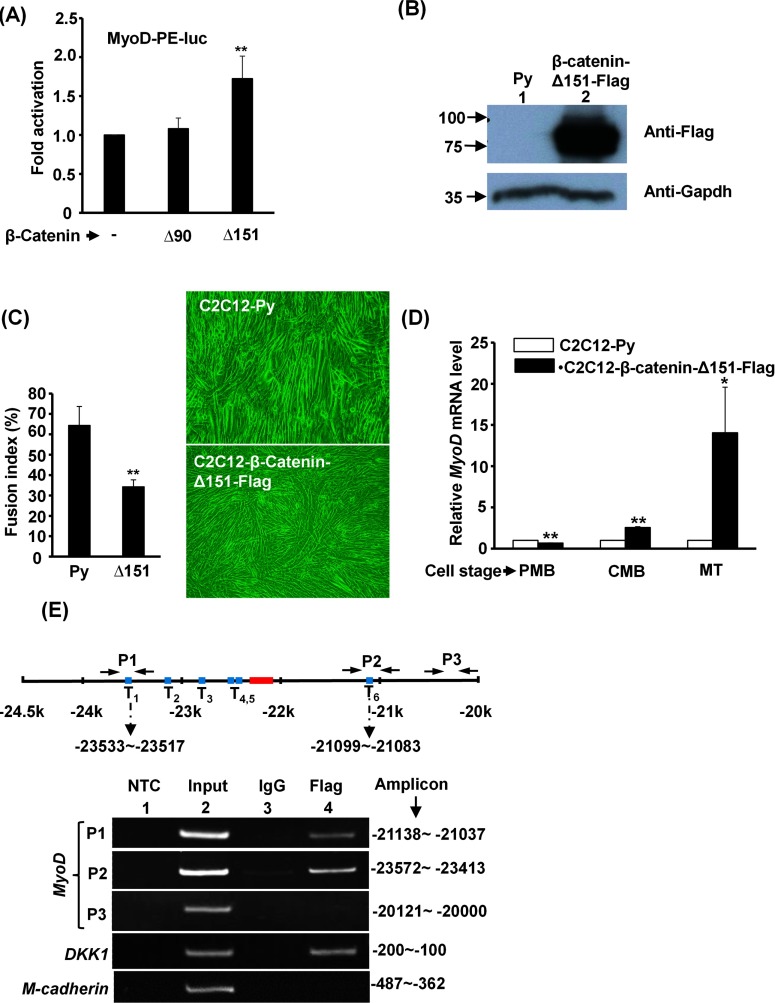
Constitutively active β-catenin enhances MyoD expression (**A**) Transient transfection of MyoD-PE-luc and β-catenin expression vectors into C2C12 cells and the luciferase activity was assayed 48 h after transfection. Δ90 and Δ151 represent N-terminally truncated β-catenin without the first 90 and 151 amino acids respectively. (**B**) C2C12 stable clones carrying either Py or Py-β-catenin Δ151–FLAG were established and their β-catenin Δ151–FLAG levels were examined by Western blotting with anti-FLAG antibody. Gapdh served as a control. (**C**) Myotube morphology and fusion index (%) after 4 days in differentiation medium. (**D**) Relative *MyoD* mRNA levels in these stable clones of various stages [proliferating myoblast (PMB), confluent myoblast (CMB) and myotube (MT)] were determined by qRT-PCR. (**E**) Binding of β-catenin Δ151–FLAG to the TCF/LEF sites in the *MyoD* gene DE region was examined by ChIP assay. Chromatin of the C2C12-β-catenin Δ151–FLAG cells at the CMB stage was precipitated with anti-FLAG antibody or control IgG and then the DNA was amplified with the indicated primer sets (P1–P3). The signals of Dickkopf-related protein 1 (DKK1) and M-cadherin serve as positive and negative controls respectively. The amplicon of each primer set is shown to the right. *P<0.05 and **P<0.01 compared with vector or vehicle control.

To explore further the roles of the canonical Wnt3a pathway in regulating MyoD expression *in vivo*, the effect of constitutively active forms of β-catenin lacking the GSK3β-targeted N-terminal regions [32] on both *MyoD* promoter activity and endogenous MyoD expression were examined. We found that β-catenin Δ151, but not Δ90, activated MyoD-PE-luc significantly (Figure 4A), therefore this active mutant was chosen to examine the regulation of endogenous MyoD by β-catenin. Surprisingly, overexpression of β-catenin Δ151–FLAG significantly reduced the fusion index (nuclei in myotube/total nuclei) of differentiated myotubes (Figures 4B and 4C). However, the expression levels of MyoD in β-catenin Δ151-overexpressed C2C12 cells of confluent myoblast (CMB) and myotube (MT) stages were higher than that in control cells of the same stages (Figure 4D), demonstrating the activation effect of the canonical Wnt signal pathway on endogenous MyoD expression.

As shown in Figure 2(C), the two flanking TCF/LEF sites, T1 (5′-TCCATTTCAAAGGCCCA-3′) and T6 (5′-TGCTTCTTTGATGCTTC-3′), seemed to play important roles in the Wnt3a response, therefore it was of interest to verify whether these two sites in the genome were targeted by β-catenin. Using a ChIP assay, we found that β-catenin Δ151–FLAG specifically bound to the T1 and T6 sites in C2C12 cells of the CMB stage (Figure 4E), confirming their participation in the *MyoD* gene response to Wnt3a intracellularly.

### A genomic fragment between the DE and the P6P in the *MyoD* gene also mediates the Wnt3a response

**Figure 5:**
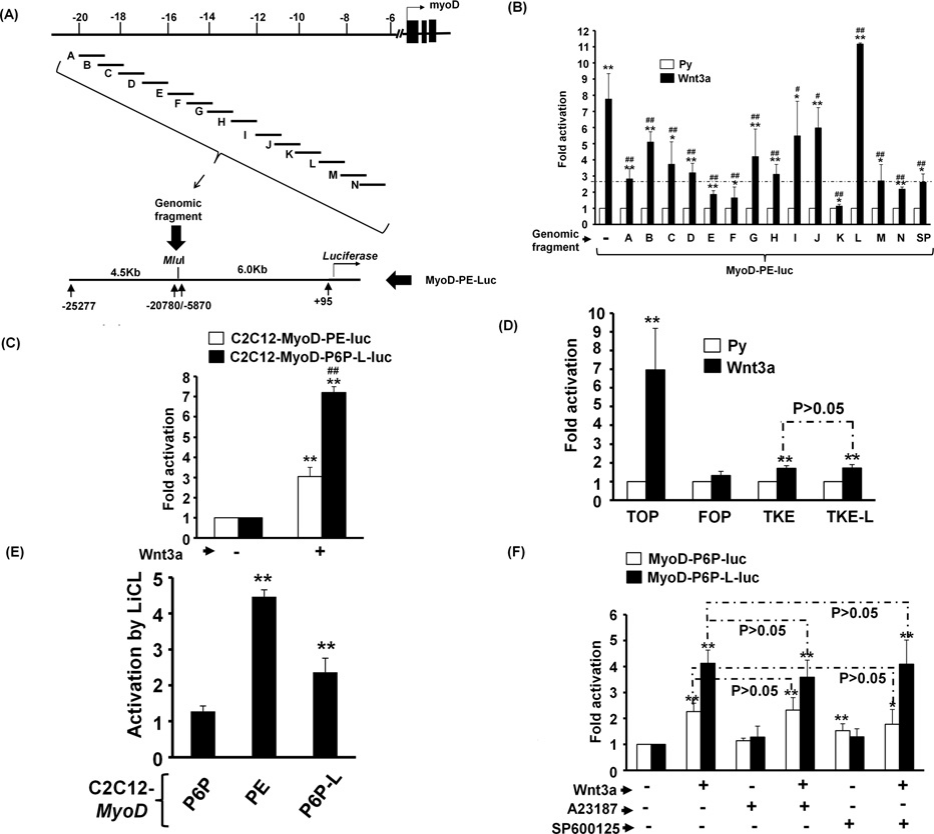
*Cis*-elements between the DE and the P6P also mediate the Wnt3a response (**A**) Genomic fragments between the DE and the P6P were inserted into the MluI site in MyoD-PE-luc and their response to Wnt3a is shown in (**B**). SP, a random vector sequence (1 kb) without known transcription factor-binding sites serves as a non-specific spacer. All reporters used were stably carried by C2C12 cells. (**C**) C2C12 stable clones carrying either MyoD-PE-luc or MyoD-6.0-L-luc were treated with Wnt3a and their luciferase activity was determined 48 h after treatment. *P<0.05 and **P<0.01 compared with vehicle (Py) control. (**D**) Transient transfection of reporters into C2C12 cells to test their response to Wnt3a as described in Figure 1(C). TOP/FOP, TOPflash/FOPflash; TKE: thymidine kinase promoter and enhancer driven reporter; TKE-L: reporter driven by L fragment plus TKE. (**E** and **F**) C2C12 stable clones carrying various *MyoD cis*-elements were treated with Wnt3a and/or its downstream pathways inhibitors or agonists to identify pathways mediating the activation of the L fragment.

Previous studies on *MyoD* gene regulation focused mostly on the P6P and the DE; the roles of *cis*-elements in other regions were seldom investigated. Whether *cis*-elements outside the P6P and the DE participate in the Wnt3a response is also an open question. The Wnt3a-targeted region in the *MyoD* gene was dissected further by inserting individual genomic fragments (approximately 1–1.5 kb each) located between the DE and the P6P into the MyoD-PE-luc reporter to mimic their location *in vivo* (Figure 5A). Surprisingly, insertion of a non-specific spacer sequence of comparable size (1 kb) reduced the Wnt3a-induced activation (Figure 5B, SP), implying that the DE functions in a distance-sensitive manner. Insertion of most intervening fragments shown a similar effect, but inclusion of the E, F, K or N fragment further reduced Wnt3a-induced activation, suggesting the existence of putative repressor-binding sites in these fragments. Of these intervening fragments, fragment L (−9489 to −7890) was the only one showing a positive effect on Wnt3a-induced MyoD-PE-luc activation and it invited more effort to decide whether extra WREs could be found in this fragment.

### Wnt3a activates the L fragment via both canonical and Pax3/Pax7-mediated pathways

The identification of extra WREs in the L fragment revealed complex transcriptional regulation of *MyoD* and pointed out the important involvement of *cis*-elements located outside the P6P and the DE regions in *MyoD* gene transcriptional activation. The L fragment was tested for its function independently of the DE, and we found that the L fragment alone could confer a strong P6P response to Wnt3a, which was significantly higher than that conferred by the DE (Figure 5C), indicating that the L fragment response to Wnt3a was strongly independent of the DE. It was of interest to know whether the L fragment acted in a promoter-specific manner. To answer this question, this fragment was cloned upstream of the thymidine kinase promoter and enhancer (TKE), an ubiquitously active transcription driver widely used for testing the effect of other *cis*-elements. We found that the L fragment lost its response to Wnt3a when it was connected to the TKE (Figure 5D), suggesting that communication with *cis*-elements in the P6P plays critical roles in its response to Wnt3a.

**Figure 6:**
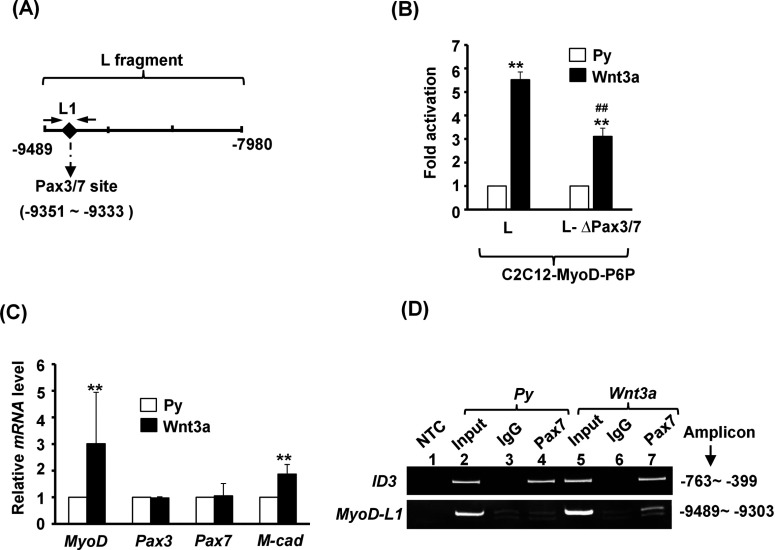
The L fragment mediates the Wnt3a response via canonical and Pax3/Pax7-mediated pathways (**A**) Schematic diagrams of the locations of the Pax3/Pax7-binding site in the L fragment. (**B**) C2C12 stable clones carrying P6P-L or P6P-L-ΔPax3/7 were treated with vehicle or Wnt3a. **P<0.01 compared with Py; ##*P*<0.01 compared with Wnt3a-treated P6P-L-luc. (**C**) The relative mRNA levels of MyoD, M-cadherin (M-cad), Pax3 and Pax7 in CMB C2C12 cells treated with or without Wnt3a were examined with qRT-PCR. The mRNA level of each gene in cells treated with Py medium was set as 1-fold. **P<0.01 compared with Py. (**D**) ChIP assay of vehicle or Wnt3a-treated C2C12 cells with IgG or anti-Pax7 antibody and the primer set (L1) shown in (**A**). The signals of the *Id3* gene serve as a positive control of the Pax7-targeted site. NTC, no template control.

The pathways involved in activating the L fragment were also examined and we found that LiCl only marginally instigated the activity of MyoD-P6P-L-luc (Figure 5E). Although the composite reporter MyoD-PE-L-luc could be activated by LiCl dose-dependently, however, this activation was not much different from that activated by Wnt3a (Supplementary Figures S1A and S1B). Bioinformatics analysis of its sequence did not find conserved (matrix similarity >0.9) TCF/LEF- or NFAT-binding sites; however, a Pax3/Pax7 target site (Pax3 and Pax7 bind identical DNA motifs [33]) were found in this region. Treatment with JNK inhibitor or Ca^2+^ ionophore had no effect on Wnt3a-activated L fragment activity (Figure 5F), suggesting that neither pathway was involved. In contrast, deletion of the putative Pax3/Pax7-targeted site (−9353 to −9334, 5′-GATGTCATGGGTACATAAT-3′) significantly reduced its activation by Wnt3a (Figures 6A and 6B), suggesting that activated Pax3/Pax7 partially mediates Wnt3a effects on the L fragment.

Using qRT-PCR, the expression of both Pax3 and Pax7 in C2C12 myoblasts was demonstrated (Figure 6C). The levels of Pax7 protein were also examined (Supplementary Figure S2), and we found further that Pax7 bound to this putative target site *in vivo*, albeit with much weaker affinity than that in the previously identified *Id3* gene [34], implying that this site is a *bona fide*, but weak, Pax3/Pax7 target site mediating the Wnt3a effect (Figure 6D). As the involvement of Pax3/Pax7 in the transduction of Wnt3a signalling has not been reported previously, the details of this novel mechanism by which Wnt3a triggers Pax3/Pax7 to activate the L fragment remains to be determined, clarification of which might shed new light on Wnt signalling transduction and activation of downstream effectors.

## DISCUSSION

### Wnt3a functions as an upstream regulator of myogenesis

Wnt3a has been shown to enhance the generation of multipotential mesendodermal (including myogenic) progenitors from embryonic stem cells and the myogenic differentiation of P19 embryonic carcinoma cells [35,36]. It is expressed not only in the neural tube, but also in the epiblast transgressing primitive streak that become the precursors of mesodermal and endodermal progenitors [37]. *Wnt3a*-null mice show normal anterior (1–7) somites, but defective posterior (after somite 7) somites, suggesting its essential role in the somitogenesis of trunk paraxial mesoderm, in which Wnt3a seems to function as a bridge linking the node/organizer derived wave front (mainly Fgf8) and the Notch pathways to regulate the periodic/cyclic epithelization of presomitic mesoderm [38]. However, whether the Wnt3a signal directly activates the expression of myogenic determination regulators, either Myf5 or MyoD, in somites has not been examined. Therefore the direct regulation of MyoD activation by Wnt3a observed in the present study is the first reporting this observation.

In adult SKM, quiescent satellite cells express undetectable or very low levels of Myf5 or MyoD, and they start to actively express MyoD upon the induction of proliferation signals, such as exercise and muscle damage. The effect of MyoD on satellite cell replenishment and their ability to regenerate damaged tissue is dose-dependent, whereby high MyoD levels repress satellite cell self-renewal and drive their myogenic differentiation or apoptosis [10]. Although previous studies have shown both positive and negative effects of Wnt3a on adult muscle regeneration [21], the observed adverse effect of Wnt3a on adult muscle regeneration is probably an artefact caused by the constitutive overdose of MyoD that was induced by Wnt3a.

The beneficial effects of Wnt3a are supported by the observation that exercise induces several Wnts in SKM, which in turn promote myogenesis by activating the proliferation and differentiation of residential satellite cells [39]. The same study also demonstrated the increased binding of β-catenin/LEF to a TCF-binding site located in the 2.7k and 2.4k regions of the *MyoD* and *Myf5* promoters respectively after voluntary exercise. Although the binding of β-catenin/LEF to this TCF-binding site was not examined in this study, this site might mediate the week activation of the MyoD-P6P-luc reporter by Wnt3a (Figure 2B). The detailed promoter analysis performed in the present study has identified two other key Wnt3a-targeted sites in the *MyoD* upstream region (Figures 4 and 6) and it will be interesting to know whether exercise-induced Wnts, including Wnt3a, can also target these sites to activate MyoD expression in satellite cells.

### The *cis*-elements regulating MyoD expression

Previous studies on the regulation of MyoD expression mainly focus on the DE and the two regulatory regions (PRR and DRR) in the P6P [13,14]. The positive-feedback regulation of MyoD is mediated by E-box P1 and P2 located in the proximal promoter [40], and an activator protein 1 (AP1) site (−342 to −32) in the same region represses MyoD activation [41]. A highly conserved core sequence of 258 bp in the distal enhancer is capable of driving LacZ expression in a spatiotemporal pattern indistinguishable from endogenous MyoD during embryogenesis; however, it is dispensable for Myf5- and Pax3-dependent regulation of *MyoD* transcription, and its deletion does not cause obvious defects in myogenesis [42,43]. The DRR enhancer is essential for *MyoD* expression in postnatal muscle and it contains a CArG element targeted by both serum-response factor (SRF) and myocyte enhancer factor 2 (MEF2) protein for initiation and maintenance of MyoD expression in satellite cells [44]. Both the core enhancer and DRR are targeted by Six1/Six4 homeoproteins for participating in the *Pax3*/*MyoD* genetic pathway [45]. Expression of MyoD in SKM can also be activated by thyroid hormone (3,3′,5-tri-iodothyronine or T_3_) and the T_3_-response element (TRE) has been located to the −337 to −309 region of the proximal promoter [46]. However, the analysis of other *cis*-elements regulating MyoD expression has been hindered by the strict requirement of both DRR and PRR in a region that extends over a sequence of 6.0 kb, i.e. the P6P, and the fact that promoters shorter than this or that lack either region show very low or undetectable activity (results not shown).

Owing to the requirement of the P6P, very few previous studies have been devoted to analysing the contribution of *cis*-elements outside the DE and the P6P in the *MyoD* gene, and, to our knowledge, the present study is the first one analysing the contribution of the intervening region between the DE and the P6P to MyoD expression. The identification of the strong response of the L fragment to Wnt3a shows the important involvement of the intervening regions in the regulation of MyoD expression. Besides, several fragments (E, F, N and K) in this region have repressed the Wnt3a-activated MyoD-PE-luc activity, implying that they contain critical negative regulatory *cis*-elements that prevent the haphazard expression of MyoD in cells of other lineages. More endeavours in the future should be devoted to analysing their targeting factors and interactions/cross-talk with other *cis*-elements in the DE and the P6P to reveal the mechanism of the tightly restricted expression of MyoD in SKM.

Moreover, although MyoD has been known for more than two decades, its induction, but not forced expression, in cells of other lineages has not been successful to turn these cells into muscle-forming myoblasts [47]. With the carefully constructed *MyoD* gene reporters set up in the present study, it will allow us to identify the direct targeting sites of various activating signals, such as retinoic acid, and repressing signals, such as Shh, in the *MyoD* gene more easily, which in turn should reveal the mechanisms governing the SKM-specific MyoD expression. The unravelled mechanisms might pave the way for reprograming cells of non-muscle lineages into MyoD-expressing myoblasts for treating degenerating muscle diseases, such as Duchenne muscular dystrophy.

## Online data

Supplementary data
