# Supplementary material for: Wnt3a signal pathways activate MyoD expression by targeting cis-elements inside and outside its distal enhancer
Source: Biosci Rep. 2015 Mar 18;35(2):e00180. doi: 10.1042/BSR20140177 (PMC4370097; doi:10.1042/BSR20140177)
Supplement: Supplementary data [file bsr035e180ntsadd.pdf]

Supplementary legends

FIGURE S1: LiCl activates MyoD-PE-L-luc dose-dependently.
C2C12-MyoD-PE-L-luc stable clones were treated with Wnt3a or LiCl (10 mM) and their luciferase activity was determined 48 hr after treatment **(A)**. Their response to increasing amount (2, 5, and 10 mM) of LiCl was also determined **(B).** **: $p<0.01$ vs. vehicle control.

FIGURE S2 Pax7 protein levels in C2C12 cells.
The levels of Pax7 protein in total lysate (50 µg) from C2C12 cells of CMB and MT stages were examined with Western blot using antibodies against Pax7 and Gapdh. Numbers to the left represent molecular weight in kilodalton (Kd).

FIGURE S1

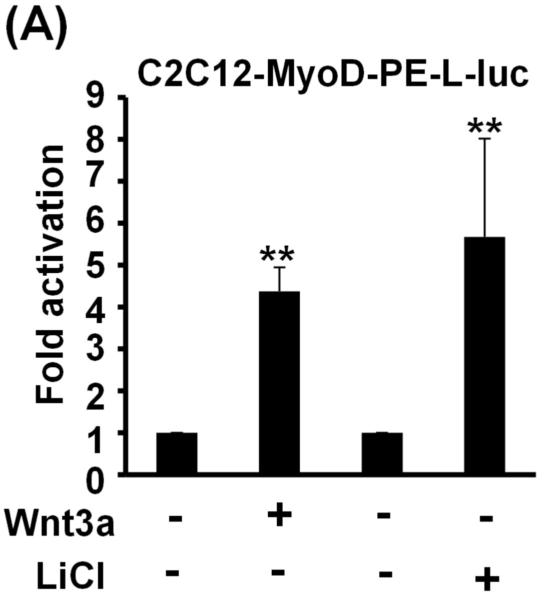

(A)
C2C12-MyoD-PE-L-luc
Fold activation
0
1
2
3
4
5
6
7
8
9
**
**
Wnt3a
-
+
-
-
LiCl
-
-
-
+

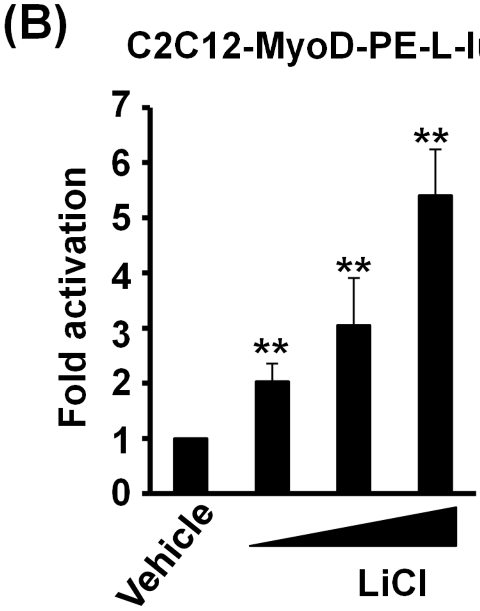

(B)
C2C12-MyoD-PE-L-luc
Fold activation
0
1
2
3
4
5
6
7
**
**
**
Vehicle
LiCl

FIGURE S2

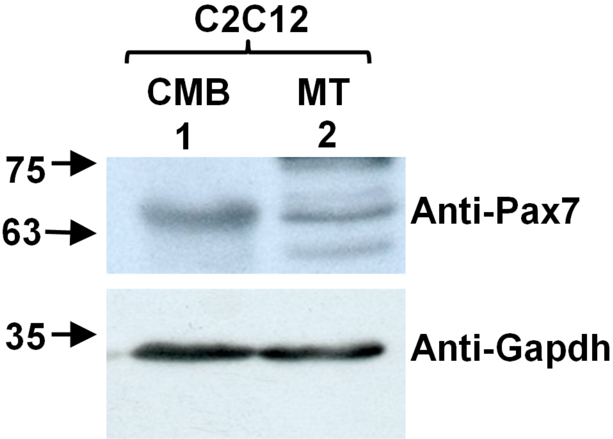

Table S1: Primer sets used for cloning.

NCU code	Clone name	Primer Sequence*	Amplicon size (bp)
026022<br>026023	*DE*	FP：5'-**cggaattc**gtgttgcccagttatcccag-3'<br>RP：5'-**cggaattc**gctggctgggtctctctagtac-3'	4497
021003<br>021004	*P6P*	FP：5'-**ggggtacc**ccagtagaggcaaagaaaggaa-3'<br>RP：5'-**ctagctagc**taggtgggttggaatgcacac-3'	2965
026022<br>039005	*DE-#1*	FP：5'-**cggaattcg**tgttgcccagttatcccag-3'<br>RP：5'-**cggaattccg**cctgcctgcctcaggcct-3'	1776
039004<br>039010	*DE-#2*	FP：5'-**cggaattcg**ctcttcacctcttc-3'<br>RP：5'-**cggaattcg**catgctcactctggc-3'	1489
039002<br>039003	*DE-#3*	FP：5'-**cggaattcg**ccaggcccttccttct-3'<br>RP：5'-**cggaattcg**agtgcacagagacggcc-3'	1491
039011<br>026023	*DE-#4*	FP：5'-**cggaattcg**agcccaggggtacatgaa-3'<br>RP：5'-**cggaattcg**ctggctgggtctctagtac-3'	1215
026022<br>039010	*DE-#5*	FP：5'-**cggaattcg**tgttgcccagttatcccag-3'<br>RP：5'-**cggaattcg**catgctcactctggc-3'	2581
026022<br>039003	*DE-#6*	FP：5'-**cggaattcg**tgttgcccagttatcccag-3'<br>RP：5'-**cggaattcg**agtgcacagagacggcc-3'	3269
039002<br>026023	*DE-#7*	FP：5'-**cggaattcg**ccaggcccttccttct-3'<br>RP：5'-**cggaattcg**ctggctgggtctctagtac-3'	2712
039004<br>039003	*DE-#8*	FP：5'-**cggaattcg**ctcttcacctcttc-3'<br>RP：5'-**cggaattcg**agtgcacagagacggcc-3'	2177
039004<br>026023	*DE-#9*	FP：5'-**cggaattcg**ctcttcacctcttc-3'<br>RP：5'-**cggaattcg**ctggctgggtctctagtac-3'	3398
039004<br>039023	*DE-#10*	FP：5'-**cggaattcg**ctcttcacctcttc-3'<br>RP：5'-**cggaattcg**ctggtacccaggagg-3'	2922
33005<br>33006	*A fragment*	FP：5'- **cgacgcgtcg** tat gta agg cta atg aga tcc cgg -3'<br>RP：5'- **cgacgcgtcg** ctg gct cag ggt ctg gga -3'	1402
33026<br>33027	*B fragment*	FP：5'- **cgacgcgt** acttgccagccaagcagcccct -3'<br>RP：5'- **cgacgcgt** gcagccctgagatccc -3'	1161

33024<br>33025	*C fragment*	FP：5’- **cgacgcgt** gggctcccagggttataa -3’<br>RP：5’- **cgacgcgt** gaaggaaggaaaggggag -3’	1381
33022<br>33023	*D fragment*	FP：5’- **cgacgcgt** ctcctccctgaacactac -3’<br>RP：5’- **cgacgcgt** ctccgctcacccccccccc -3’	1316
33020<br>33021	*E fragment*	FP：5’- **cgacgcgt** caggacacaggcagggca -3’<br>RP：5’- **cgacgcgt** acttaggaggtggaggt -3’	1340
26027<br>26028	*F fragment*	FP：5’-**cgacgcgtc** ccatcatatcatctaagcta -3’<br>RP：5’-**cgacgctc** ccacaattcccagattctcttgc -3’	1116
33018<br>33019	*G fragment*	FP：5’- **cgacgcgt** ccctgcctccctctttccc -3’<br>RP：5’- **cgacgcgt** ccatctctgtccccatcc -3’	1366
33016<br>33030	*H fragment*	FP：5’- **cgacgcgt** gat gct ccc aga ctc cca -3’<br>RP：5’- **cgacgcgt** acactcaccatgtaccc -3’	1254
33014<br>33015	*I fragment*	FP：5’- **cgacgcgt** ggg tac atg gtg agt gtc -3’<br>RP：5’- **cgacgcgt** gtg ggt ggg gtg ggg tgg g -3’	1290
33011<br>33012	*J fragment*	FP：5’- **cgacgcgt** cccaccccaccccacccac -3’<br>RP：5’- **cgacgcgt** accctccaagtatccc -3’	1240
033034<br>033043	*K fragment*	FP：5’-**cgacgcgt**CCTCCTCTGTGCTGCCTTA-3’<br>RP：5’-**cgacgcgt**GGTTTCCTTTCTAAGTGC-3’	715
33007<br>33008	*L fragment*	FP：5’- **cgacgcgt** gcaggcatgaaggacatg -3’<br>RP：5’- **cgacgcgt** ggtgttagtcattctgggg -3’	1510
33003<br>33004	*M fragment*	FP：5’- **cgacgcgtcg** cct gcc ttt cac acc tga tg -3’<br>RP：5’- **cgacgcgtcg** aca cat gtc tca tcc -3’	1174
33001<br>33002	*N fragment*	FP: 5’- **cgacgcgtc** ggt acc tga tgt gtg cct gg -3’<br>RP: 5’- **cgacgcgtcg** tat gct cta aat aaa atc tat tc -3’	1142
039020<br>039021	*β-Catenin-Δ90*	FP: 5’-**cgcggatcc**atggctcagagggtcc-3’<br>RP: 5’-**cgcggatcc**caggtcagtatcaaaccag-3’	2079
039026<br>039021	*β-Catenin-Δ151*	FP: 5’-**cgcggatcc**atggcaattcctgagct-3’<br>RP: 5’-**cgcggatcc**caggtcagtatcaaaccag-3’	1896

***: linkers and restriction sites are shown in bold.**

FP: forward primer

RP: reverse primer

Table S2: Primer sets used for amplifying gene in RT-PCR

NCU code	Gene name	Primer Sequence	Amplicon size
00324<br>00325	*MyoD*	FP：5'-ggctacgacaccgcctacta-3'<br>RP：5'-gttctgtgtcgcttagggat-3'	204 bp
006012<br>006013	*Myogenin*	FP：5'-ccagtgaatgcaactcccacagc-3'<br>RP：5'-agacatatcctccaccgtga-3'	166 bp
001042<br>001043	*Gapdh*	FP：5'-cctctggaaagctgtggcgt-3'<br>RP：5'-ttggcaggtttctccaggcg-3'	190 bp
006001<br>006002	*M36b4*	FP：5'-ggcagcatttataaccctgaagtg-3'<br>RP：5'-cggacaccctccagaaagc-3'	73 bp

FP: forward primer

RP: reverse primer

Table S3: Primer sets used for amplifying genes in CHIP assay

NCU Code	Name	Primer sequence	Amplicon size (bp)
039047<br>039048	*MyoD-P1*	FP: 5'- **ccc ctt ccc ctc acc at**- 3'<br>RP: 5'- **cgg tga gtc cct cca ag** - 3'	102
039049<br>039050	*MyoD-P2*	FP: 5'- **ctt ggt tgt gta tcc tgc t**- 3'<br>RP: 5'- **tca aat tcc tgt agc c** - 3'	160
039051<br>039052	*MyoD –P3*	FP: 5'- **tga gca agt gtt gcc cag tta t**- 3'<br>RP: 5'- **gac caa cct cct atg tct a**- 3'	121
039041<br>039042	*DKK1*	FP: 5'- **cca gcg ctt tga aat ccc at** - 3'<br>RP: 5'- **acc gcg gct gcc ttt ata cc**- 3'	100
029124<br>029125	*M-cadherin*	FP: 5'-**ctc tcc cgg gca gcc ctc tg**-3'<br>RP: 5'-**gcg cat gcg tcc tgc tcc cga t** -3'	126
039024<br>039057	*MyoD-L1*	FP: 5'- **cgg aat tcg cag gca tga agg aca tg** - 3'<br>RP: 5'- **tgg aca cct cac acg ctc**- 3'	187
039058<br>039059	*Id3*	FP: 5'- **ccg ggc ata cat tta gtt cct** - 3'<br>RP: 5'- **tct ctc tct cct ctc tct ctc aa**- 3'	365

FP: forward primer

RP: reverse primer
